# Sleep and circadian regulation of DNA repair and retrotransposons in neuronal genome stability and neurodegenerative disease mechanisms

**DOI:** 10.3389/fnins.2026.1865440

**Published:** 2026-09-15

**Authors:** Abhas Rajhans, Ella Kye, Vedant Balu, David L. Glanzman, Ketema N. Paul

**Affiliations:** 1 Ketema Paul Lab, University of California Los Angeles, Department of Integrative Biology and Physiology, Los Angeles, CA, United States; 2 David Glanzman Lab, University of California Los Angeles, Department of Integrative Biology and Physiology, Los Angeles, CA, United States

**Keywords:** circadian rhythms, DNA repair, epigenetic regulation, genome instability, LINE-1, neurodegenerative disease, retrotransposon, sleep

## Abstract

Sleep and wake are fundamental brain states that coordinate diverse physiological processes required for long-term health and survival. Recent research in molecular neuroscience and chronobiology has begun to clarify how sleep–wake cycles and circadian timing intersect with genomic maintenance mechanisms in the nervous system. In this Review, we summarize current evidence that sleep and circadian rhythms temporally regulate neuronal genome stability, with a particular focus on how they modulate neuronal retrotransposon activity. We discuss circadian clock-dependent regulation of DNA repair pathways, the contribution of sleep to chromosomal integrity and chromosome dynamics, and emerging data on temporally-dependent retrotransposon activity across the sleep–wake cycle. Taken together, the studies we review suggest that sleep may provide a permissive state during which neurons engage genome-protective processes, including up-regulation of DNA repair factors, changes in chromatin organization, and silencing of retrotransposon activity. We further propose that these mechanisms may be relevant to neurodegenerative disorders, although much of the current evidence linking sleep and circadian disruption to retrotransposon dysregulation, genomic instability, and cognitive decline remains preclinical or correlative in humans.

## 1 Introduction

The relationship between sleep and wakefulness is central to cellular and neuronal health. During waking periods, deep-layer pyramidal neurons of the frontal cortex in mice, mushroom body neurons in *Drosophila*, and dorsal pallium, telencephalon, rhombencephalon neurons in zebrafish engage in high metabolic and synaptic activity. Neuronal activity is required for cognitive processing but also leads to the accumulation of DNA damage, reactive oxygen species, and metabolic stress in neural cells (Bellesi et al., 2016; Zada et al., 2019, 2021).

During sleep, these deleterious effects are reversed. In zebrafish, genes like *Parp1* are up-regulated at onset of sleep, but also initiate DNA repair processes. *Parp1* inhibition reduces NREM sleep duration and intensity in mice, suggesting that DNA repair may have functional links to low periods of cognitive load during sleep (Wang et al., 2021; Zada et al., 2019, 2021).

Circadian rhythms synchronize sleep–wake cycles with environmental time, aligning DNA repair, metabolism, and gene expression in neurons with daily behavioral states (Sancar et al., 2010). Recent work in *Drosophila* and mouse models demonstrates that DNA damage increases during wakefulness in response to novel environment exposure or learning events, and that repair processes are most active during sleep, when cellular conditions are optimized for genomic maintenance (Bellesi et al., 2016; Zada et al., 2021; Wang et al., 2021).

Retrotransposons like LINE-1 and Alu are ubiquitous in mammalian neurons. Recent studies in human and cynomolgus monkey mesenchymal progenitor cells show that LINE-1 activation is controlled by DNA methylation and non-canonical circadian regulatory proteins, including BMAL1, further suggesting a role for epigenetic regulation in sleep and circadian cycles (Faulkner et al., 2009; Liang et al., 2022; Mukherjee et al., 2016; Vasieva et al., 2017).

Here we review how sleep–wake cycles maintain neuronal genome stability. We present current evidence supporting the integration of retrotransposon regulation with sleep–wake cycles, and discuss the significance of these genomic processes for neurodegenerative disease. The studies we report span multiple model organisms and human cohorts but reveal important clues about how the system works as whole.

## 2 Methods

### 2.1 Inclusion criteria

Our literature review is composed of: (1) peer-reviewed articles examining relationships between sleep/circadian regulatory mechanisms and genomic maintenance/repair mechanisms; (2) studies investigating how DNA repair processes fluctuate during sleep–wake cycles; (3) research on retrotransposon activity and regulation in neurons and neural tissues; (4) investigations of circadian control over DNA damage response pathways; (5) clinical and experimental studies linking sleep disruption to genomic instability in neurons; (6) studies investigating DNA damage in neurodegenerative disorders; and (7) clinical studies examining retrotransposon activity and/or DNA damage and/or sleep–wake cycles.

### 2.2 Exclusion criteria

We excluded: (1) studies focusing exclusively on sleep/circadian mechanisms; (2) research limited to non-mammalian systems without translational relevance; (3) correlational studies lacking mechanistic insights; (4) studies that do not examine or are irrelevant to neurons and neural systems; and (5) case reports and review articles with non-clinical and/or non-original data.

### 2.3 Operationalized terms

#### 2.3.1 Sleep fragmentation

In cited studies, fragmentation is induced by forced awakenings at regular intervals, mechanical or sensory stimuli, or scored by actigraphy as decreased uninterrupted sleep epochs.

#### 2.3.2 Sleep deprivation/restriction

Sleep deprivation: Complete prevention of sleep for a defined period (e.g., 6–24 h), usually via gentle handling or environmental disturbance. Sleep restriction: Reduction of total sleep time per cycle, limiting access to sleep opportunities.

#### 2.3.3 Circadian disruption/misalignment

Perturbation of normal circadian rhythms through phase shifts, forced activity/rest schedules, or exposure to light during the habitual night period.

#### 2.3.4 DNA damage

Assessed by molecular markers (γ-H2AX expression, comet assay for DNA strand breaks), up-regulation of DNA repair genes, or increased frequency of chromosomal aberrations.

#### 2.3.5 Epigenetic silencing/DNA methylation

Quantified by bisulfite sequencing, methylation-specific PCR, or methylation microarray. Reported as locus-specific or global cytosine methylation percentages.

#### 2.3.6 NREM/REM cycle/sleep architecture

Defined by EEG and behavioral scoring; NREM (non-rapid eye movement) and REM (rapid eye movement) cycles identified by characteristic waveforms and muscle atonia. Sleep architecture refers to distribution, duration, and sequence of NREM and REM across the sleep period. In mice, cycles typically last 10–20 min; in humans, 60–90 min.

#### 2.3.7 Clinical interventions

CBT-I: Cognitive Behavioral Therapy for Insomnia, delivered by sleep psychologist over multiple sessions, targeting maladaptive sleep behaviors and cognition. Light therapy: Exposure to bright light at specific times to shift or entrain circadian phase, typically administered in morning hours.

Figure generation and analysis was completed via Google Gemini and Almaa Labs (usealmaa.com).

## 3 Results

### 3.1 Sleep, circadian rhythms, and genomic stability

#### 3.1.1 Circadian coordination of DNA repair

Circadian rhythms orchestrate cellular processes, including metabolism, transcription, and genomic maintenance, in a tightly coordinated manner. These rhythms are linked to environmental light–dark cycles (Sancar et al., 2010). The mammalian molecular clock operates through interdependent transcription-translation feedback loops, primarily involving the genes *Clock, Bmal1, Period,* and *Cry*, which regulate both core clock output and help direct DNA repair activity. In murine and rodent models, the timing of DNA repair is governed by rhythmic expression of these core clock proteins. The CRY1 protein, in particular, engages in time-of-day–dependent interactions with the Ataxia Telangiectasia and Rad3-related (ATR)-mediated DNA checkpoint response, establishing specific periods during which neuronal DNA repair capacity is elevated (Kang and Leem, 2014). Cryptochrome modulates ATR activation under conditions of metabolic and genotoxic stress, enhancing the resolution of DNA double-strand breaks in neurons at discrete phases of the sleep–wake cycle (Kang and Leem, 2014). It is important to note that the majority of studies that form the foundation for circadian regulation of DNA repair have been conducted in vertebrate species.

Recent work in primate cells has identified a potential role for BMAL1 in neuronal genome defense. Using human and cynomolgus monkey-derived cell systems, Liang et al. (2022) showed that BMAL1 loss is associated with elevated markers of genomic instability, and that BMAL1 can physically interact with LINE-1 sequences and nuclear lamina proteins (Liang et al., 2022). In this study, BMAL1-deficient primate neurons exhibited elevated LINE-1 mobilization and increased markers of genomic instability. Molecular genetic analysis and gene expression profiling were employed to demonstrate that disruptions in circadian clock function, such as BMAL1 loss, directly impair retrotransposon repression and lead to subsequent DNA damage in the central nervous system. These findings support a model in which BMAL1 contributes to epigenetic silencing of LINE-1 under normal circadian conditions, such that clock disruption may permit increased retrotransposon activity and DNA damage. However, these data derive from a limited number of cellular and tissue studies, and extrapolation to *in vivo* human neuronal circuits and neurodegenerative disease pathogenesis remains provisional.

#### 3.1.2 Sleep-dependent DNA repair and chromosome dynamics

Sleep provides a conserved period of low synaptic load for genomic maintenance and DNA repair in post-mitotic neurons. Experimental studies in zebrafish have demonstrated that neuronal chromosome dynamics increase markedly during sleep, facilitating the repair of DNA double-strand breaks (DSBs) accumulated during wakefulness (Zada et al., 2021). By expressing H2B-mCherry fusion protein in zebrafish neuronal and two non-neuronal cell subtypes, they calculated the rate and magnitude of chromatin movements using time-lapse visual tracking and computational image analysis. During wakefulness, chromosome movements in neurons were minimal, and DSBs accumulated over time; in contrast, sleep dramatically increased chromosome dynamics specifically in neurons (but not in two other tested cell types), which correlated with a reduction in DSBs: DNA damage plus recovery sleep approximately doubles chromosome dynamics in control neurons. This increase in dynamics during sleep is consistently associated with upregulation of Rad52 and Ku80 foci. The authors infer that sleep-associated chromatin remodeling—operationalized as increased chromosome dynamics—mechanically enables laminar proteins to access and bind damaged DNA in post-mitotic neurons.

They also blocked sleep with pharmacological inhibitors, and found that DSBs persisted. Conversely, recovery sleep after forced wakefulness reduced the accumulated DSBs (Zada et al., 2021). In mature, non-dividing neurons, homologous recombination and non-homologous end joining repair are less active and access to break sites is constrained by stable chromatin architecture, but sleep increases the number of Ku80-EGFP and DsRed-Rad52 nuclear foci specifically during nighttime sleep and rebound sleep. These sleep-dependent, neuroprotective changes in chromosome mobility enable more efficient access for DNA repair machinery.

Other studies in mice using sleep-restriction and behavioral manipulation protocols have shown that neuronal populations restore genomic stability during sleep by upregulating DNA damage response genes (Bellesi et al., 2016). Their research demonstrated that sleep deprivation not only increases DNA damage but also impairs the expression of genes critical for repair pathways such as homologous recombination and non-homologous end joining (Bellesi et al., 2016). In mice, exploration during waking periods increased cortical DNA DSBs, and subsequent sleep restored DSB levels to baseline. Sleep also accelerated the repair of irradiation-induced DSBs and upregulated DNA repair genes (*Brcc3, Tipin, Chek2, Nek1, Parp1, and Sirt1*) (Bellesi et al., 2016). Complementary work in Drosophila mushroom body neurons has shown that post-learning sleep is accompanied by changes in the expression of RNA processing and translation-related genes, consistent with broader sleep-dependent regulation of chromatin and transcriptional machinery in memory circuits (Li et al., 2024). These findings support a primary role for sleep architecture in supporting neuronal genomic integrity.

The relationship between Rapid Eye Movement (REM), Non-Rapid Eye Movement (NREM) sleep, and neural processes is complex. Narwade et al. (2017) performed selective REM sleep deprivation (REMSD) via the flower pot method and compared gene expression profiles in REMSD rats vs. controls; they found that in REMSD rats, genes related to histone modifications (*HIST1H1C*, *H2BC8*, *etc.*), as well as to synaptic transmission and memory (*NMDA Receptor Subunit 2B, Oxytocin, Hypocretin/Orexin*) were downregulated (Narwade et al., 2017). In addition, the genes *Atp1b2, Dbh, Syt2, Npas4,* and *Egr1* were upregulated in REMSD rats, reflecting a response to increased stress/arousal (*Dbh, Npas4, etc.*) and perhaps enhanced compensatory memory consolidation due to loss of REM sleep (*Egr1, Syt2*) (Narwade et al., 2017).

Together, these results suggest that sleep serves as an active period for DNA repair in brain cells, counterbalancing the accrued synaptic load due to metabolic activity during wakefulness. Expression of repair genes is enhanced during sleep, while induction of plasticity-related genes is reduced, prioritizing DNA repair and low synaptic load (during NREM sleep) and promoting the restoration of cellular homeostasis (Bellesi et al., 2016; Narwade et al., 2017; Zada et al., 2019, 2021).

#### 3.1.3 Sleep–wake cycle and genomic instability in neurodegenerative diseases

Chronic sleep disruption leads to changes in genomic stability and promotes neurodegenerative processes in preclinical models. In transgenic mouse models of Alzheimer’s disease (AD), sustained sleep deprivation is associated with altered expression of core circadian clock genes, increased tau phosphorylation, and greater amyloid deposition (Niu et al., 2022). Chronic sleep deprivation (CSD) impaired learning and memory, increased amyloid-β plaque deposition, elevated Aβ42/40 ratios, and enhanced tau phosphorylation in the retrosplenial cortex, locus coeruleus, and suprachiasmatic nucleus (Niu et al., 2022). These effects were accompanied by altered expression of the *Bmal1*, *Clock*, and *Cry1* genes in circadian rhythm-related nuclei and the pineal gland, particularly in AD mice (Niu et al., 2022). CSD also elevated tyrosine hydroxylase levels in the locus coeruleus, indicating heightened noradrenergic activity (Niu et al., 2022). Single-cell multiomic studies in human Alzheimer’s disease brains have now revealed cell-type and region-specific epigenomic erosion, including loss of chromatin compartmentalization and heterochromatin stability, suthat disruption of temporal and epigenetic organization undermines neuronal resilience and contributes to neurodegenerative progression (Liu et al., 2025). These findings outline correlations between sleep loss and neuropathology, but further mechanistic studies are required to delineate direct causal pathways.

Building on this relationship between sleep disruption and genomic instability, recent investigations have explored how chronic alterations in circadian and sleep processes can directly accelerate pathological changes in the brain. Sleep disturbances are observed across several neurodegenerative conditions, including AD and Parkinson’s disease (PD) (Rigat et al., 2023; Hunt et al., 2022). In PD mouse use models, early circadian rhythm disruption coincides with increased neuronal vulnerability and impaired genomic maintenance. Neurons are sensitive to oxidative stress, and this sensitivity escalates with metabolic demand during wakefulness. Data from mice with *Bmal1* gene knock-out in neurons and astrocytes demonstrated excessive oxidative stress in the brain and increased neurodegeneration linked to reduced transcription of redox-protective genes (Musiek and Holtzman, 2016). The mice also display accelerated age-dependent synaptic degeneration, increased astrogliosis, and heightened neuroinflammation (Musiek and Holtzman, 2016). Additional experiments showed that loss of BMAL1 protein in astrocytes leads to increased oxidant production and severely impaired antioxidant gene expression in the brain (Musiek and Holtzman, 2016). Recent work has extended these observations to neuroimmune cells, showing that disruption of intrinsic microglial circadian clocks alters cytokine production and exacerbates neuroinflammatory responses, further linking circadian dysfunction to inflammatory mechanisms (Lu and Kim, 2025). However, the impact of sleep deprivation varies across cell-types and regions: emerging spatial transcriptomic datasets further demonstrate that sleep deprivation induces pronounced, region-specific changes in gene expression across the hippocampus, neocortex, and other brain areas, highlighting heterogenous molecular consequences of disrupted sleep across neural circuits (Vanrobaeys et al., 2023).

There is a bidirectional relationship between circadian clock gene dysfunction and neurodegeneration. Cermakian et al. (2011) measured the expression and day-night rhythmicity of three core clock genes—*Per1*, *Per2*, and *Bmal1*—via qPCR in the pineal gland, cingulate cortex, and bed nucleus of the stria terminalis (BNST), in postmortem samples from 27 AD patients and 31 controls at different times of death, the collection of samples at various circadian phases proxied as circadian timing (Cermakian et al., 2011). They detected rhythmicity for all three genes, but AD brains showed altered amplitude, phase, or synchrony compared to gene expression in controls. In controls, the expression of *Bmal1* and *Per2* generally peaked in antiphase (i.e., ~12 h apart), as classically expected. In AD brains, the amplitude and phase of the rhythms of the genes’ expression were disrupted, and inter-regional and inter-gene synchrony was reduced (Cermakian et al., 2011). In control pineal glands and BNSTs, *Bmal*1 had significant 24-h rhythmicity; peaking at night in controls, while in AD brains, showing significant 24-h rhythmicity, often with altered phase in all three regions compared with controls (Cermakian et al., 2011). *Bmal1* phase/synchronicity of expression between regions was disturbed in the BNST of AD samples (*Bmal1* and *Per2* phases): while nearly antiphase in controls, the phases were much closer together in AD patients (Cermakian et al., 2011). These molecular disruptions parallel and likely contribute to the severe disturbances of sleep, activity, and cognition in AD patients. Taken together, these studies support the idea that circadian clock genes and the brain’s sleep–circadian system are important for maintaining temporal organization of neural function between sleep and wake states and for buffering oxidative and metabolic stress (Cermakian et al., 2011; Musiek and Holtzman, 2016; Zada et al., 2021). However, while impaired clock gene synchronization is associated with disrupted antioxidant defenses, metabolism, protein homeostasis, and cognitive function, the extent to which specific components such as circadian clock-influenced LINE-1 regulation causally drive human neurodegenerative disease remains an open question.

Sleep and wake are tightly integrated through the action of core circadian clock genes such as *Bmal1* that promote sleep onset but may also regulate canonical (sleep onset) and non-canonical (retrotransposon regulation, DNA repair, memory consolidation) processes during sleep (Cermakian et al., 2011; Faulkner et al., 2009; Liang et al., 2022; Mukherjee et al., 2016; Vasieva et al., 2017). The restorative and protective roles—DNA repair, synaptic growth, reactive oxygen species (ROS) clearing through cerebrospinal fluid (CSF)—executed during sleep, in turn, influence neuronal resilience and cognitive performance in the next wake period (Bellesi et al., 2016; Cermakian et al., 2011; Hunt et al., 2022; Rigat et al., 2023).

Rather than viewing sleep disturbances solely as downstream consequences of neurodegeneration, the available literature increasingly supports the possibility that sleep and circadian dysfunction may contribute to neural decline and interact bidirectionally with disease processes. However, this framework should still be regarded as a working model: although preclinical studies support mechanistic links among clock dysfunction, oxidative stress, DNA damage, and neuronal vulnerability, many human data remain associative and do not yet establish a definitive causal pathway between disturbed sleep or circadian timing and neurodegenerative progression. Future clinical studies should therefore test whether targeting sleep and circadian processes can modify molecular and clinical trajectories of neurodegenerative disease.

### 3.2 Retrotransposon regulation and temporal control

#### 3.2.1 Retrotransposon activity in neural systems

Retrotransposons are mobile genetic elements that propagate via an RNA intermediate and form a substantial proportion of mammalian genomes (Faulkner et al., 2009). These elements are grouped into long terminal repeat (LTR) and non-LTR classes; of the non-LTR class, long interspersed nuclear element-1 (LINE-1) is the only active autonomous retrotransposon in mammals, while short interspersed elements (SINEs), such as Alu, depend on LINE-encoded proteins for mobilization. SINEs hijack LINE-1 reverse transcriptase, while LINE-1 can self-reproduce and is compatible with SINE transcripts (Belancio, 2015; Faulkner et al., 2009). Faulkner and colleagues established that these elements share replication via reverse transcription but differ in protein-coding capacity and regulatory control (Faulkner et al., 2009). LINE-1 encodes proteins for open reading frame 1 (ORF1p) and open reading frame 2 (ORF2p), which are necessary for autonomous retrotransposition, whereas SINEs require helper proteins for movement (Belancio, 2015). Mechanistically, current primate data indicate that BMAL1 represses LINE-1 by acting as a heterochromatin stabilizer at LINE-1-containing regions: BMAL1 binds LINE-1 sequences and nuclear lamina/heterochromatin-associated proteins, promotes maintenance of H3K9me3-marked chromatin, and thereby keeps LINE-1 transcriptionally silent; when BMAL1 is lost, heterochromatin destabilizes, LINE-1 transcripts increase, and downstream cGAS–STING signaling and cellular senescence are activated (Liang et al., 2022).

When LINE-1 is derepressed, its ORF2p endonuclease and reverse transcriptase drive target-site–primed retrotransposition, which introduces new insertions and associated nicks and breaks in genomic DNA; potentially allowing BMAL1-dependent epigenetic silencing and PIWI–piRNA–guided methylation to prevent LINE-1-mediated genomic instability in neural and germline tissues (Faulkner et al., 2009; Kuramochi-Miyagawa et al., 2008; Liang et al., 2022).

Mukherjee et al. (2016) used RT-PCR to analyze LINE-1 ORF2; they found that LINE-1 elements are transcribed in the hippocampus of four-month-old rats, even without any apparent stress or experimental manipulation, implicating retrotransposons in neuronal diversity and genome dynamics (Mukherjee et al., 2016). In silico analysis showed that LINE-1 insertion loci were near genes that are themselves actively transcribed in the hippocampus (*Nkain3, Mtif2, Atf6, Rabgap1l, Dock1, Lin7a,* and *Cyp20a1*), suggesting that LINE-1 activity alters the expression of neighboring genes (Mukherjee et al., 2016). “Liang et al. (2022) used CRISPR/Cas9 to generate BMAL1 knockout (KO) human and cynomolgus monkey cells and examined heterochromatin stability, LINE-1 transcription, and markers of cellular senescence. *In vitro* and *in vivo* assays showed that BMAL1 KO was associated with reduced H3K9me3 staining, chromatin loosening, and increased LINE-1 transcript abundance in several tissues, and chromatin immunoprecipitation indicated BMAL1’s physical interaction with LINE-1 sequences and nuclear lamina proteins (Liang et al., 2022). These findings suggest that BMAL1 participates in circadian regulation of LINE-1 silencing and chromatin organization, consistent with evolutionary evidence that retrotransposon regulation is integrated into mammalian clock systems (Vasieva et al., 2017). Nonetheless, the BMAL1–LINE-1 relationship has so far been characterized in a restricted set of primate cellular and tissue models, and its direct contribution to human neurodegenerative disease mechanisms remains to be established. Recent syntheses of transposable element biology in human neurons redirect the narrative, emphasizing that LINE-1 and other elements participate in shaping neuronal transcriptomes, long non-coding RNA and microRNA networks, and activity-dependent gene regulation, rather than having purely deleterious function (Chesnokova et al., 2022).

#### 3.2.2 Evolutionary recruitment of transposable elements

Growing evidence suggests that over the course of evolution, retrotransposition has come to play important roles in behavior and cognition in mammals (Glanzman 2024). In support of this idea, Vasieva et al. (2017) identified and mapped primate-specific retrotransposon sequences using SVA (hominid-specific non-LTR retrotransposon SINE VNTR Alu) loci in the human genome (Hg19) from the UCSC Genome Browser (Vasieva et al., 2017). SVA loci are classified as ancient (A, B, C), intermediate (D), and young (E, F, and F1) subfamilies. They analyzed brain genomic DNA and chromatin accessibility, determining that behavioral characteristics and complex thought processes, like memory consolidation, are encoded by genes containing younger SVA elements, while older SVA elements were associated with genes impacting primitive behaviors (e.g., climbing). They found *Creb1* (cAMP Responsive Element Binding Protein 1) is involved in both vertical and spatial learning and place preference, and is associated with an SVA E I (young SVA E-type element). Similarly, *PSEN1* (*Presenilin-1*) is associated with an SVA F (young SVA F-type element) element and plays a role in social order recognition, memory and social behavior. PSEN1 is part of the gamma-secretase complex, important in processing amyloid precursor protein. The cleavage of PSEN1produces amyloid-beta peptides implicated in AD. Huntingin (*HTT*) was also found to have numerous SVA A and B-type (ancient SVA-type elements) intragenic insertions, related to learning, social behavior, and motor and spatial learning. Vasieva et al. (2017) also found that SVA insertions were overrepresented in genes tied to neuropsychiatric conditions, including AD, schizophrenia, bipolar disorder, epilepsy, intellectual disabilities (including X-linked mental disabilities), and movement and mood disorders (Vasieva et al., 2017). Complementary investigations conducted genome-wide analyses in the mammalian genome (Takahashi, 2017). They found that RNA polymerase II recruitment and transcription initiation, and specific histone modifications occur with circadian rhythm across most expressed genes. Circadian clock proteins modulate chromatin via interaction with chromatin “remodelers” such as histone acetyltransferases (p300, CBP), methyltransferases (MLL1), and histone deacetylases (SIRT1), regulating promoter accessibility and global transcriptional rhythms, indicating that transposable elements contribute significantly to circadian regulatory regions (Takahashi, 2017).

Evolutionary analyses in mammalian systems carried out by Judd et al. (2021) showed that specific transposable element families have been co-opted as regulatory enhancers for circadian transcription factors (Judd et al., 2021). This study revealed that these transposable enhancer elements contribute substantially (approximately 14%) to circadian transcription factor binding sites in mammalian genomes. While this conclusion is based on a single study, Vasieva’s and Takahashi’s work provide corroborating evidence for the regulatory integration of transposons into mammalian genomic architecture.

#### 3.2.3 Environmental and temporal influences

Environmental factors influence retrotransposon regulation through effects on circadian rhythms and sleep. Light exposure at night disrupts melatonin production and has been linked to genomic instability via effects on retrotransposon activation (Belancio, 2015). de Haro et al. (2014) exposed mammalian cells to melatonin and compared LINE-1 retrotransposon activity in cells with and without functional melatonin receptors; they found that cells with functional melatonin receptors had lower rates of LINE-1 retrotransposon activity, which suggests that melatonin receptor-environmental light interactions influence genome stability in mammalian cells (de Haro et al., 2014).

Experimental models of shift work and circadian disruption demonstrate wide-ranging effects on DNA methylation (Bollati et al., 2010; Jansen et al., 2019). Bollati et al. (2010) examined blood samples from shift workers and day workers and found that the chronic circadian misalignment associated with shift work was associated with lower methylation at Alu and LINE-1 sequences (Bollati et al., 2010). They operationalized “job seniority,” measured as employment in years, and found that each additional year of shift work was associated with lower Alu, Interferon gamma (IFN-γ), and tumor necrosis factor alpha (TNF-α) methylation rates. Jansen et al. (2019) found that sleep duration is inversely related to LINE-1 methylation in boys, supporting the idea that DNA remodeling and repair occurs in response to stimulus/environmental exposure during waking periods (Jansen et al., 2019). Thus, activity during waking periods appears to have direct impacts on sleep-phase processes, including inflammatory, stress, and DNA modification pathways. Retrotransposon activity may have distinct acute and chronic responsibilities in response to stress or chronic sleep misalignment/circadian disruptions, as suggested by the lower methylation rates for Alu and inflammatory genes caused by stress, sleep misalignment, and circadian disruptions.

### 3.3 Molecular mechanisms and epigenetic control

#### 3.3.1 DNA methylation, chromatin modifications, and epigenetic silencing

DNA methylation operates as a primary silencing mechanism for retrotransposons, particularly LINE-1, and serves as an indicator of global genomic methylation status (Bollati et al., 2010). Environmental disturbances such as shift work have been shown to alter methylation patterns across the genome, potentially impacting retrotransposon regulation and long-term DNA stability (Bollati et al., 2010; Jansen et al., 2019). Sleep quality and duration correlate with methylation variation and may have sex-specific effects (Jansen et al., 2019). Jansen et al. performed actigraphy-based sleep recording and bisulfite pyrosequencing (for *LINE-1*, *H19, PPARA, HSD11B2*; DNA damage marker, imprinted metabolism-regulator gene, metabolic gene, stress hormone regulator gene, respectively), and found that, in boys, longer sleep duration (top quartile) was associated with lower LINE-1 methylation compared to quartile 3 (Q3) (Jansen et al., 2019). In girls, both longer and shorter sleep duration were associated with higher LINE-1 methylation, when compared to LINE-1 methylation in Q3 of sleep duration (Jansen et al., 2019). These findings suggest that changes in DNA methylation form an important molecular link between sleep patterns and metabolic and/or stress-related health risk during adolescence/pubertal development.

Studies involving long-term sleep disruption and circadian misalignment, such as shift work models, indicate persistent changes in epigenetic regulation through repetitive elements (Bollati et al., 2010; Jansen et al., 2019). Moreover, experimental findings in mice have shown that sleep loss and irregular circadian timing cause measurable differences in cognitive function (Bellesi et al., 2016; Hunt et al., 2022).

Bellesi et al. (2016) explored this phenomenon by exposing in mice and across the whole *Drosophila* brain to various sleep and wake periods (6-h periods) interventions and measuring DSBs in the prefrontal cortex (Bellesi et al., 2016). Cohorts of mice and flies were given various treatments: stimulation that caused the animals to stay awake—novel environment exposure for mice, and social engagement for flies: (“enriched wake”); awake with no stimulation (“wake”); and sleep. Each of the wake cohorts also had cohorts allowed to sleep before they were harvested (“wake and sleep”). In addition, there was a cohort of mice engaged in a wake session while running on a treadmill (“exercise wake”). Animals engaged in any form of learning (enriched wake) had significantly higher DSBs than wake mice/flies, while exercise wake mice had similar DSB levels to wake mice (Bellesi et al., 2016). Additionally, the mice/flies in the enriched wake condition—given a 6-h sleep period—exhibited reduced DSB levels, similar to those in the wake/exercise wake groups (Bellesi et al., 2016). Their findings demonstrate that learning during waking periods may increase prefrontal cortex DSBs, which subsequent sleep periods then repair (Bellesi et al., 2016). These findings, together with emerging evidence of melatonin-influenced LINE-1 regulation, support the idea that sleep and circadian patterns contribute to retrotransponsition/epigenetic modifications during sleep, and may be a response to activities performed in previous wake periods.

P-element Induced WImpy testis (PIWI) proteins may play a central role in retrotransposon silencing through coordination of DNA methylation and chromatin modifications (Kuramochi-Miyagawa et al., 2008). PIWI RNAs (piRNAs) are small RNAs that bind to PIWI proteins and give them sequence specificity. In mice, miwi-like (MILI), and mouse piwi 2 (MIWI2) are key PIWI proteins that carry piRNAs that are complementary to LINE-1 and Intracisternal A Particle (IAP) retrotransposons. Kuramochi-Miyagawa et al. (2008) showed in PIWI-knockout (PIWI-KO) mice models, that MILI or MIWI2 KO disrupts normal *de novo* methylation in regulatory regions of LINE-1 and IAP retrotransposons of mice primordial germ cells (PGCs), which causes de-repression (activation) of retrotransposon expression, as evidenced by higher levels of LINE-1 and IAP mRNA transcripts in *MILI* and *MIWI2* KO mice. Methylation-sensitive Southern and bisulfite data in neonatal germ cells confirmed CpG hypomethylation in both *MILI* and *MIWI2*-KO germ cells in LINE-1 and IAP regulatory regions (Kuramochi-Miyagawa et al., 2008). MILI and MIWI2 bind to repeat-associated small RNAs called piRNAs. piRNAs produced in fetal testes target retrotransposons, guiding DNA methylation at specific genomic sites. piRNA expression is severely reduced in *MILI* or *MIWI2-*KO germ cells. The work of Kuramochi-Miyagawa et al. (2008) cites prior work confirming that all DNA methylation is globally erased early in PGC development, and that the quiescent phase involves PGCs pausing cell division to begin *de novo* methylation. That is, new methylation is added to specific DNA regions, including LINE-1 retrotransposon promoters. In *MILI* or *MIWI2-*KO mice, those piRNA populations disappear, and *de novo* methylation at LINE-1 and IAP is lost (Kuramochi-Miyagawa et al., 2008). This PIWI-dependent methylation is essential for silencing LINE-1 expression so that transposons do not become active in the developing germline (Kuramochi-Miyagawa et al., 2008).

PIWI-mediated silencing machinery operates through small RNA-guided recognition of transposable elements followed by recruitment of methylation and chromatin remodeling enzymes (Kuramochi-Miyagawa et al., 2008). While the PIWI pathway is best characterized in germline cells, recent evidence points to its presence in somatic tissues, including neurons (Kuramochi-Miyagawa et al., 2008; Liang et al., 2022; Mukherjee et al., 2016). In Aplysia, a neuronal Piwi–piRNA complex guides DNA methylation to the CREB2 promoter and is required for long-term synaptic facilitation through its repression of a key memory-suppressor gene (Nandi et al., 2016; Landry et al., 2013). In mammals, PIWI and piRNA are expressed in neurons and participate in silencing retrotransposons, suggesting that Piwi–piRNA directed epigenetic control is relevant for memory-related genomic stability (Landry et al., 2013; Rajasethupathy et al., 2012). In mature neurons, PIWI–piRNA complexes have been shown to guide DNA methylation at specific promoters (such as CREB2 in Aplysia) to regulate long-term synaptic facilitation and memory, and by analogy to their established role in germline LINE/LTR retrotransposon silencing, are hypothesized to contribute to maintaining neuronal genome stability by repressing transposable elements and modulating activity-dependent gene expression. Similarly, in regenerative flatworms such as *Schmidtea mediterranea*, the PIWI proteins SMEDWI-2 and SMEDWI-3, together with piRNAs, are required for silencing transposable elements in somatic stem cells and for preserving genome integrity during regeneration (Palakodeti et al., 2008). However, definitive evidence for PIWI-dependent retrotransposon control in mammalian post-mitotic neurons remains limited (Kuramochi-Miyagawa et al., 2008; Landry et al., 2013; Rajasethupathy et al., 2012).

Relative to neurons, glial cells—particularly astrocytes—contribute to genomic maintenance largely through circadian and redox support: astrocytic BMAL1 loss drives excessive oxidant production, reduced antioxidant gene expression, astrogliosis, and neuroinflammation, thereby indirectly increasing neuronal vulnerability to DNA damage and impairing genome stability, whereas most of the retrotransposon and sleep-dependent chromatin remodeling mechanisms we discuss are characterized in neurons themselves (Musiek and Holtzman, 2016; Zada et al., 2021). The conservation of PIWI–piRNA pathways across germline, regenerative somatic tissues, and neuronal systems underscores their broader importance for transposon control and genomic stability, and warrants future research in mammalian neuronal and glial cells.

Current evidence indicates that LINE-1 activity in neural systems occupies both causative and reactive roles in neurodegenerative conditions. In primate models, loss of BMAL1 function leads to chromatin loosening, derepression of LINE-1, and downstream genomic instability and senescence, positioning LINE-1 activation as a proximal driver of neuronal DNA damage when circadian gatekeeping fails (Liang et al., 2022; Musiek and Holtzman, 2016). At the same time, epidemiological and experimental data show that LINE-1 methylation and expression are highly responsive to upstream sleep and circadian disturbances (chronic shift work, adolescent sleep fragmentation, melatonin suppression) suggesting that LINE-1 is also a dynamic effector that reacts to environmental and behavioral stressors (Bollati et al., 2010; de Haro et al., 2014; Jansen et al., 2019). Baseline hippocampal transcription of LINE-1 in healthy rats, and its proximity to actively transcribed neuronal genes, further supports a model in which retrotransposon activity contributes to normal neuronal genomic plasticity but becomes pathogenic when regulatory systems are disrupted (Mukherjee et al., 2016; Vasieva et al., 2017). Thus, in the context of neurodegeneration, LINE-1 appears to participate in a bidirectional loop: circadian and sleep dysregulation derepress LINE-1, whose mobilization then aggravates DNA damage and disease progression [2].

In summary, retrotransposons emerge as both direct drivers of genomic instability—via derepressed LINE-1/SVA mobilization causing heterochromatin disruption and DNA damage when circadian and PIWI/piRNA silencing fail—and sensitive secondary markers of broader epigenetic dysregulation, as reflected by their methylation and expression tracking sleep, circadian, and environmental stress. However, it is important to note that LINE-1 is primarily investigated in pre-clinical models, with little investigation into longitudinal LINE-1 characterization in humans/primate models at present.

## 4 Limitations and conclusions

Several limitations should guide interpretation of the current literature. First, although circadian and sleep disruption are consistently associated with genomic instability and neurodegenerative phenotypes, causality remains difficult to establish because many available data are correlational or derived from complex perturbation models in which multiple pathways are altered simultaneously (Cermakian et al., 2011; Musiek and Holtzman, 2016; Niu et al., 2022; Rigat et al., 2023). Second, cross-study comparison is complicated by substantial methodological heterogeneity, as retrotransposon activity has been inferred using distinct readouts including LINE-1 methylation, ORF2 transcription, H3K9me3-associated heterochromatin status, and insertion mapping, which do not capture identical biological processes (Jansen et al., 2019; Mukherjee et al., 2016; Liang et al., 2022; Vasieva et al., 2017). Third, there are too few longitudinal human studies directly linking sleep or circadian disruption to retrotransposon activity and neuronal genomic instability (Bellesi et al., 2016; Liang et al., 2022; Zada et al., 2019, 2021). Fourth, neuron-specific epigenomic datasets in human sleep research remain limited, forcing inference from blood-based methylation studies, whole-brain or mixed-cell analyses, and non-neuronal tissues that may not fully capture cell-type-specific regulation in the human nervous system (Bollati et al., 2010; Jansen et al., 2019; Liang et al., 2022). Finally, markers of genomic instability may be influenced by aging, inflammation, oxidative stress, metabolic dysfunction, and medication exposure, all of which frequently co-occur with sleep and neurodegenerative disorders and can confound interpretation of retrotransposon and DNA damage measurements (Musiek and Holtzman, 2016; Hunt et al., 2022; Rigat et al., 2023; Sobreira-Neto et al., 2023).

It is also important to note that the integrative framework developed in this review draws on diverse experimental systems, including zebrafish and Drosophila models of sleep-dependent DNA repair and chromosome dynamics, rodent studies of cortical double-strand breaks and clock gene disruption, primate BMAL1–LINE-1 epigenetic studies, and human shift-work, adolescent sleep, and postmortem circadian gene expression cohorts (Bellesi et al., 2016; Zada et al., 2019, 2021; Musiek and Holtzman, 2016; Cermakian et al., 2011; Bollati et al., 2010; Jansen et al., 2019; Liang et al., 2022). These systems differ substantially in neuronal architecture, lifespan, sleep architecture, and retrotransposon repertoires, and many mechanistic findings—such as sleep-dependent chromosome dynamics in zebrafish or BMAL1-mediated LINE-1 silencing in primate cells—have not yet been directly confirmed in human neurons *in vivo*. As such, the comparative approach is best viewed as generating a convergent, hypothesis-generating picture of how sleep and circadian rhythms might interface with genome maintenance, rather than as establishing a single conserved pathway across all species.

## 5 Implications

Therapeutic strategies for neurodegenerative disease increasingly target both the wake and sleep phases of the circadian cycle. During wakefulness, neurons engage in behaviors and plasticity that are metabolically taxing and lead to DNA damage accumulation. Under optimal conditions, sleep follows and activates the molecular machinery responsible for DNA repair, retrotransposon silencing, and restoration of genomic stability (Bellesi et al., 2016; Musiek and Holtzman, 2016; Wang et al., 2021; Zada et al., 2019, 2021). Clinical interventions such as cognitive behavioral therapy for insomnia (CBT-I) and light-based circadian modulation aim to reinforce healthy sleep architecture and rhythmic transitions between wake and sleep. CBT-I, used to minimize excessive wakefulness and optimize conditions for neuronal recovery, can provide clinical benefit by taking advantage of the relationship between synaptic load during wake and appropriate sleep architecture function. CBT-I and related circadian interventions may help restore behavioral and temporal alignment between wake and sleep states, and are therefore plausible candidates for indirectly supporting genomic homeostasis; however, direct evidence that these interventions modify neuronal DNA repair, retrotransposon silencing, or genome instability markers in humans is still limited.

REM sleep behavior disorder (RBD) is characterized by REM sleep without atonia (RWA), motor behaviors and vocalizations during REM sleep, associated with dreams and nightmares (Sobreira-Neto et al., 2023). RBD is distinct from other sleep disorders, such as sleep obstructive apnea (SOA) (Sobreira-Neto et al., 2023). Sobreira-Neto et al. (2023) have reviewed clinical studies of RBD, and find that dream enactment and similar CBT techniques help reduce RBD symptoms in patients. They also reviewed current literature on pharmaceutical treatment for RBD; they found few double-blind randomized clinical trials for RBD treatment, but report that melatonin (instant release, dose range 3–15 mg) appears to be helpful in reducing the dream-acting frequency and RWA in clinical trials in males. Low-dose (0.25-1 mg) clonazepam is also a common prescription for RBD; however its side effects include cognitive impairment and excessive drowsiness, among others. They note that clonazepam produces adverse effects in SOA patients; this highlights the necessity for accurate sleep disorder diagnosis for appropriate pharmaceutical treatment, as global treatment for similar disorders may produce disparate effects. Clinical trials using drugs such as cannabidiol in PD patients with RBD, Nelotanserin for RBD patients, and 5-hydroxytryptophan for RBD patients, yielded negative results, showing no difference in RBD symptom control between groups (Sobreira-Neto et al., 2023).

The research presented above highlights the importance of sleep structure, particularly the cyclical transitions between NREM and REM phases, for efficient genomic maintenance. Evidence from animal models shows that fragmentation of sleep disrupts DNA repair and epigenetic silencing pathways, and links sleep quality directly to neuronal genome integrity (Zada et al., 2021; Jansen et al., 2019). Strategies that restore natural sleep–wake patterns and circadian timing—whether through behavioral, pharmacological, or chronobiological approaches—offer promise for both prevention and mitigation of genomic instability in at-risk neurological populations. It is also important that clinicians remain sensitive to the temporally specific nature of the sleep state. They should aim to protect REM/NREM sleep in patients to improve sleep quality and increase sleep quantity/consistency. Clinicians should also diagnose sleep/circadian disorders with care, as interventions for similarly presenting disorders may have adverse effects on patients.

From a translational standpoint, the mechanistic data reviewed above justify the need for pre-clinical models to develop candidate biomarkers and intervention targets to sharpen clinical strategies beyond generalized sleep and circadian optimization. For instance, peripheral and tissue-specific measures of repetitive element methylation (e.g., LINE-1/Alu methylation in blood, and H3K9me3-associated heterochromatin status at retrotransposon loci) may offer tractable biomarkers for monitoring epigenetic silencing capacity and retrotransposon regulation (Bollati et al., 2010; Jansen et al., 2019; Liang et al., 2022). Chronotherapeutic approaches—such as timed light exposure, melatonin or melatonin receptor agonists, and behavioral scheduling—may exert protective effects in part by stabilizing BMAL1–CRY1–ATR signaling axes, reinforcing circadian gating of DNA repair and LINE-1 repression in neurons and astrocytes (Sancar et al., 2010; Kang and Leem, 2014; Musiek and Holtzman, 2016; Liang et al., 2022; de Haro et al., 2014). More targeted molecular strategies could eventually include pharmacologic modulation of PARP1-dependent sleep and DNA repair pathways, enhancement of PIWI–piRNA-mediated retrotransposon silencing, or restoration of clock gene synchrony across neural circuits (Kuramochi-Miyagawa et al., 2008; Bellesi et al., 2016; Zada et al., 2019, 2021; Musiek and Holtzman, 2016). The potential to integrate biomarkers and mechanistic targets into therapeutic interventions provides a strong rationale for development of sophisticated pre-clinical models combining neuronal DNA repair, retrotransposon activity, and neurodegenerative risk.

At present, this translational promise should be interpreted cautiously, because most supporting evidence is inferred from preclinical models or peripheral biomarkers rather than from trials demonstrating direct effects on neuron-specific genomic maintenance mechanisms in humans

## 6 Conclusion

The evidence reviewed here supports a model in which wake and sleep are associated with distinct but complementary processes relevant to neuronal genome maintenance, including DNA damage accumulation during wakefulness and enhanced DNA repair and chromatin remodeling during sleep. Emerging findings further suggest that circadian regulation and retrotransposon silencing may intersect with these processes, but the degree to which these components form a unified causal network in human neurodegeneration remains unresolved. Thus, the current literature supports a biologically plausible and increasingly testable framework linking sleep–wake dynamics, circadian regulation, retrotransposon biology, and neuronal genome stability. As therapies evolve to target sleep quality and circadian alignment, this framework may help guide future approaches for studying and potentially mitigating neurological disease risk.
