# Supplementary material for: Sleep and circadian regulation of DNA repair and retrotransposons in neuronal genome stability and neurodegenerative disease mechanisms
Source: Front Neurosci. 2026 Sep 15;20:1865440. doi: 10.3389/fnins.2026.1865440 (PMC13621125; doi:10.3389/fnins.2026.1865440)

**Figure A. Sleep-Circadian Regulation of Neuronal Genome Stability: proposed integrative model**

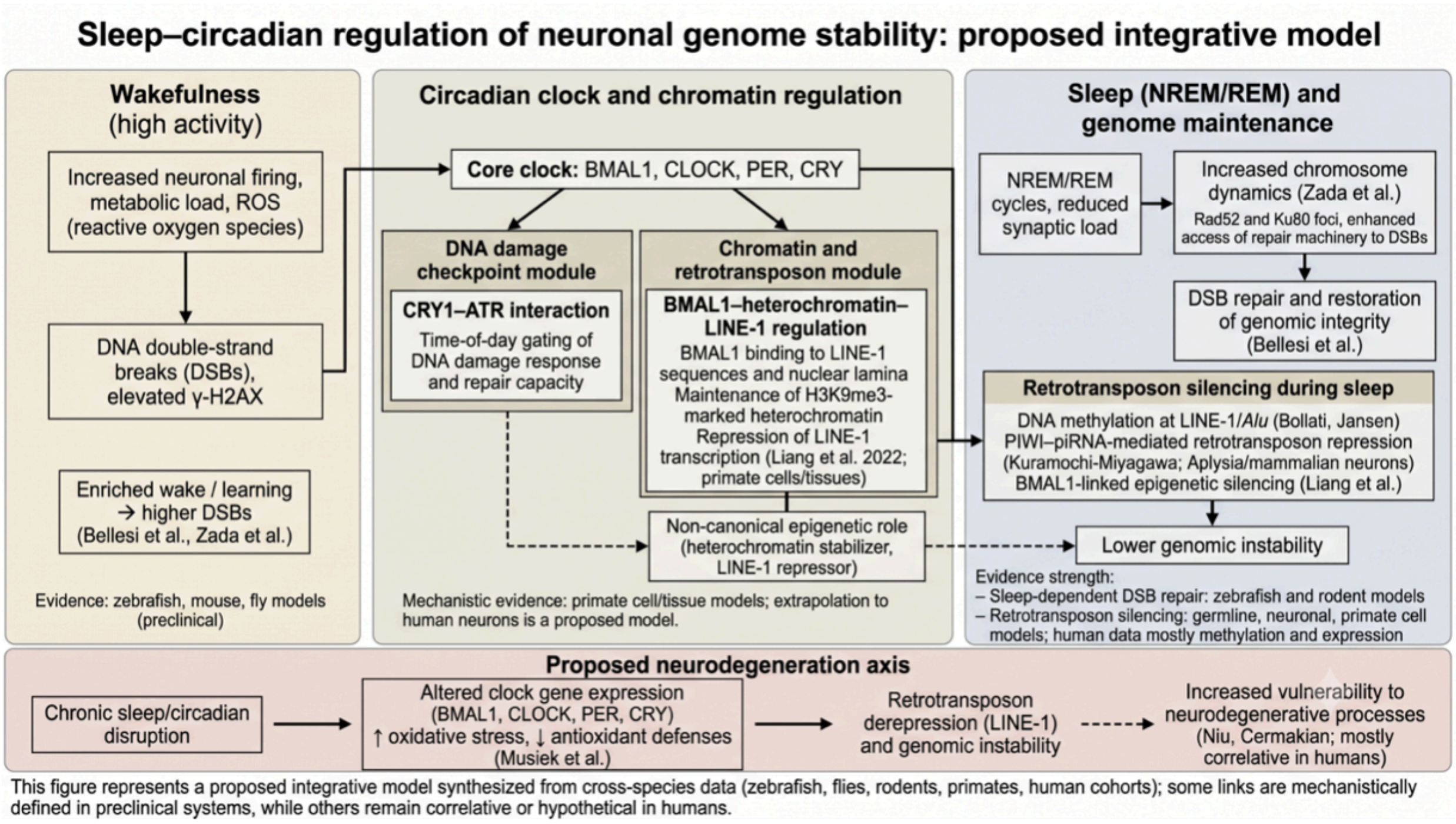

Supplement: Supplementary file 1 [file Image_1.pdf]
