# Supplementary material for: Sleep and circadian regulation of DNA repair and retrotransposons in neuronal genome stability and neurodegenerative disease mechanisms
Source: Front Neurosci. 2026 Sep 15;20:1865440. doi: 10.3389/fnins.2026.1865440 (PMC13621125; doi:10.3389/fnins.2026.1865440)
Supplement: Supplementary file 2 [file Image_2.pdf]

Figure B. Cross-species evidence supporting sleep-circadian-genome stability mechanisms.

Cross-species evidence supporting sleep–circadian–genome stability mechanisms

Legend: ● Mechanistic evidence (causal/pathway-level) ○ Correlative/associative evidence ◌ Hypothesized / extrapolated (limited direct data)

	Zebrafish	*Drosophila*	Rodents (mice/rats)	Primates / cell lines	Humans (clinical/cohort/postmortem)
**Sleep-dependent DNA damage and repair**	Sleep ↑ chromosome dynamics; DSB repair (Zada et al.) ●	Enriched wake→ DSBs; sleep normalizes (Bellesi et al.) ●	Wake/exploration ↑ cortical DSBs; sleep restores; DNA repair genes upregulated (Bellesi et al.) ●	Limited direct sleep–DSB data ◌	Indirect associations (sleep loss, DNA damage markers) ○
**Circadian clock–DNA damage response**	General clock–repair coordination; limited mechanistic detail ◌	Clock involvement in stress/repair (preclinical) ○	CRY1–ATR interaction; circadian gating of DNA checkpoint (Kang et al.) ●	BMAL1–heterochromatin; LINE-1 repression (Liang et al.) ●	Clock gene rhythmicity/disruption in AD brain (Cermakian et al.) ○
**Retrotransposon activity and regulation**	Limited direct retrotransposon data ◌	Transposon regulation via small RNAs (conceptual link) ○	LINE-1 transcription in hippocampus; insertion near active genes (Mukherjee et al.) ●	BMAL1 KO → chromatin loosening, ↑ LINE-1 transcripts, genomic instability (Liang et al.) ●	Shift work, sleep duration → *Alu*/LINE-1 methylation changes (Bollati, Jansen) ○
**Neurodegenerative phenotypes / molecular pathology**	Sleep and chromosome dynamics; neurodegeneration not directly modeled ◌	Sleep/circadian disruption affects neural function (preclinical) ○	Sleep deprivation in AD models → ↑ amyloid, tau; *Bmal1* KO → oxidative stress, neurodegeneration (Niu, Musiek) ●	BMAL1 deficiency → aging phenotype, senescence in progenitor cells (Liang et al.) ○	Sleep/circadian disturbances associated with cognitive decline, AD/PD symptoms; clock gene disruptions in AD brain (Rigat, Hunt, Cermakian)

● Mechanistic evidence (causal/pathway-level) ○ Correlative/associative evidence ◌ Hypothesized / extrapolated (limited direct data)

Evidence levels highlight that many mechanistic links are defined in zebrafish, Drosophila, rodent, and primate cell/tissue models, whereas human data are largely associative (methylation, clock gene expression, clinical symptoms). This matrix emphasizes that the integrated model is hypothesis-generating across species rather than a fully proven, established mechanism.
